# Enhancing cryopreservation performance in semen quality of Bali bulls (*Bos sondaicus*) using tris-nano-liposome extender

**DOI:** 10.5455/javar.2026.m1038

**Published:** 2026-06-22

**Authors:** Muhammad Gunawan, Tulus Maulana, Hikmayani Iskandar, Johana Dian Rahayu, Raden Iis Arifiantini, Ni Wayan Kurniani Karja, Mohamad Agus Setiadi, Ekayanti Mulyawati Kaiin, Syahruddin Said, Dedi Junian, Marianto Syam, Achmad Setiono, Anny Amalia, Akbar Akbar, Taufik Ridwan Musafak

**Affiliations:** 1 Graduate School of Reproductive Biology, School of Veterinary Medicine and Biomedical Sciences, IPB University, Bogor, Indonesia; 2 Research Center for Applied Zoology, National Research and Innovation Agency (BRIN), Jl. Raya Jakarta-Bogor, Cibinong, Bogor, Indonesia; 3 Division of Reproduction and Obstetrics, School of Veterinary Medicine and Biomedical Sciences, IPB University, Bogor, Indonesia; 4 Riau Regional Artificial Insemination Center, Jl. Tenayan No. 1, Tenayan Raya, Pekanbaru, Indonesia; 5 Singosari Artificial Insemination Center, Desa Toyomarto, Singosari, Malang, Indonesia

**Keywords:** Bali bull, cryopreservation, sperm quality, tris-egg-yolk, tris-nano-liposome

## Abstract

**Objectives:** Post-thaw semen quality in Bali bulls is largely determined by the extender used during cryopreservation. Conventional tris egg yolk–based extenders (TEY) are widely used in bovine semen freezing; however, their effectiveness may be limited by variability in biochemical composition and potential biosecurity concerns. Tris-nano-liposome–based extenders (TNL) have recently emerged as a promising alternative due to their membrane-stabilizing properties and improved cryoprotective efficiency. This study aimed to evaluate the effectiveness of TEY and TNL extenders in maintaining post-thaw semen quality in Bali bulls.

**Materials and Methods:** Semen samples from nine Bali bulls were assessed for macroscopic characteristics, microscopic parameters, and kinematic traits using a computer-assisted sperm analysis (CASA) system. Correlation analysis, principal component analysis (PCA), and partial least squares–discriminant analysis (PLS-DA) were performed to explore parameter relationships and distinguish responses between extenders.

**Results:** Both extenders maintained satisfactory post-thaw sperm quality, with no significant differences (*p* > 0.05) observed in total motility, viability, membrane integrity, acrosome integrity, and DNA fragmentation. The kinematic parameters remained within optimal ranges, indicating preserved progressive and curvilinear movement patterns. Multivariate analyses identified motility and membrane integrity as the primary contributors to variation in semen quality. Furthermore, the TNL extender produced more stable and homogeneous responses than TEY.

**Conclusions:** The TNL extender demonstrated cryoprotective performance comparable to that of the conventional TEY extender while offering advantages in clarity, biosecurity, and compositional consistency. These findings indicate that TNL represents a promising alternative extender for bovine semen cryopreservation.

## 1. Introduction

Among the most important native Indonesian bovine genetic resources is the Bali cattle (*Bos sondaicus*), which can be distinguished based on its large population size, resistance to parasites, and adaptability to the tropics. These characteristics, in addition to the ability of the Bali cattle breed to produce quality beef and its superior reproductive performance compared to other breeds in the region, highlight the importance of the breed in the national development of the bovine population in Indonesia. The Bali cattle breed has been formally identified as a native Indonesian breed based on the Decree of the Minister of Agriculture No. 325/Kpts/OT.140/1/2010 and has been found to play a critical role in the national development of the beef sector in the country [1]. In addition to its importance in the national development of the Indonesian bovine population. The Bali cattle breed has been identified as contributing to the sustainability of Indonesia’s livestock sector as a whole [2, 3].

Artificial insemination (AI) plays a pivotal role in enhancing livestock reproductive efficiency, minimizing the transmission of sexually transmitted pathogens, and improving the genetic and population management of cattle herds [4]. Semen quality is a fundamental of the success of AI programs [5]. Hence, it is crucial to have efficient cryopreservation techniques to maintain the structural integrity and functionality of sperm cells, which can then be used to their maximum potential. The advantage of cryopreservation is that it is possible to store frozen semen for long periods if it is maintained at proper temperatures using liquid nitrogen [6]. However, cryopreservation imposes significant stress on sperm cells, including ice crystal formation, osmotic imbalance, and oxidative damage, all of which compromise structural integrity and post-thaw function [7]. Semen extenders protect sperm cells by providing substances that help preserve sperm integrity [8]. Commonly, a freezing extender used for semen cryopreservation is made up of cryoprotectants such as glycerol, dimethylformamide (DMF), or dimethyl sulfoxide (DMSO), which are used to protect sperm cells during the freezing process [9].

TEY has long been incorporated as a key component of conventional semen extender media due to its high concentrations of proteins and lipids, which contribute to the protection of spermatozoa during cryopreservation and thawing processes [10]. The low-density lipoprotein fraction of egg yolk has been found to be effective in reducing the adverse effects of cold shock on spermatozoa by providing stability to the sperm plasma membrane. Nevertheless, the use of egg yolk has been associated with a number of limitations. The use of egg yolk has been found to affect the microscopic evaluation of spermatozoa, thereby affecting the accuracy of computer-assisted sperm analysis. The use of egg yolk extender has also been found to affect the evaluation of critical parameters of sperm motility. In addition, the use of egg yolk extender has been found to affect biosecurity, owing to the potential of egg yolk to transmit diseases such as avian influenza. The use of egg yolk extender has also been found to increase the risk of microbial contamination [11].

Plant-based extenders hold promise to address the hazards linked to the use of animal-derived materials, such as bacterial contamination, allergic responses, and ethical issues in semen processing. The use of plant-based materials is also in line with the concept of environmental sustainability since it reduces the use of animal materials and may lower the ecological footprint of their processing. Within the framework of the above, TNL extender is a significant breakthrough in cryopreservation techniques. Liposomes are microscopic vesicles made of a phospholipid bilayer that can encapsulate bioactive molecules [12]. Nanoliposomes, having a smaller size and greater reactivity on their surfaces, have been found to be beneficial in the improvement of the freeze-thawing process by offering protection to the sperm cells from damage due to cryoinhibition. The addition of nanoliposomes to the extender medium is found to improve the quality of thawed sperm and thereby increase the efficiency of artificial insemination [13]. However, the efficacy of TNL extenders for use in Bali bull semen is not scientifically tested and proven, and the available data is limited. Hence, the present study aimed to investigate the use of TNL and TEY extenders in Bali bulls attending two AI centers in Indonesia.

## 2. Materials and Methods

### 2.1. Ethical approval

The study was carried out from May 2024 to February 2025 at the Singosari National AI Center and the Riau Regional AI Center, Indonesia. Ethical approval for the study was granted by the ethical clearance committee of the National Research and Innovation Agency (BRIN), under approval number 166/KE.02/SK/07/2024.

### 2.2. Preparation of extenders

The chemicals employed in this study were procured from Sigma-Aldrich and Merck. The Tris-egg yolk 20% extender (TEY), commonly used at Singosari AIC and Riau RAIC, included Tris (hydroxymethyl-aminomethane), citric acid, raffinose (Singosari AIC)/fructose (Riau RAIC), glycerol, antibiotics (penicillin and streptomycin), and egg yolk. The tris-nanoliposome (TNL) extender was crafted using Tris buffer, which consisted of Tris (3.09 gm), citric acid (1.73 gm), and fructose (1.27 gm), all dissolved in 100 ml of double-distilled water (ddH_2_O). The antibiotic blend comprised gentamicin (500 µg/ml), tylosin (100 µg/ml), lincomycin (300 µg/ml), and spectinomycin (600 µg/ml). Glycerol (7% v/v) acted as a cryoprotectant, and nanoliposomes were added at a final concentration of 10 mg/ml.

### 2.3. Semen collection

Fresh semen samples from nine Bali bulls were used: four from the Singosari AIC and five from the Riau RAIC. Semen collection was carried out in the morning using an artificial vagina according to the standard operating procedures of the centers. Immediately after collection, the ejaculates were transported to the laboratory for macroscopic and microscopic examinations in a controlled environment. Only ejaculates showing >70% initial motility were used in the experiment to ensure the use of high-quality semen for further analysis.

### 2.4. Freezing procedure

Frozen semen production at both AI centers was conducted in accordance with the standard operating procedures (SOPs) with the Indonesian National Standard (SNI 4869-1:2021) for frozen semen production. At Singosari AIC, ejaculated semen extended with the TEY extender was processed using a two-step dilution process, followed by equilibration at 5°C for 24 h, and then proceeded to printing, filling, sealing, and freezing. In contrast, at Riau AIC, semen dilution using TEY was conducted in a single step with equilibration at 5°C for 4 h before cryopreservation. For both AI centers, semen extended with the TNL extender involved a single-step dilution at 35–37°C, followed by printing, filling, sealing, a 4-h equilibration, and freezing.

### 2.5. Evaluation of frozen semen

Frozen semen was thawed in a water bath at 37°C for 30 sec, gently dried, and cut at both ends to expel the contents into pre-warmed microtubes at 37°C. Subsequent evaluations included acrosome total and progressive motility, viability, morphology normality, plasma membrane integrity, acrosomal integrity, and DNA fragmentation. Furthermore, sperm motility was assessed using a CASA system. For each evaluation, 5 μl of semen was loaded onto a pre-warmed glass slide, covered with a coverslip, and examined on a heated microscope stage maintained at 37°C. Motility parameters were obtained from four microscopic fields per sample, with 300–400 spermatozoa analyzed using the AndroVision^®^ software (Minitüb, Tiefenbach, Germany) on a heated stage maintained at 37°C.

Sperm motility was evaluated using a Computer-Assisted Sperm Analysis (CASA) system (AndroVision^®^, Minitüb, Tiefenbach, Germany). A 5 μl aliquot of semen was placed onto a pre-warmed glass slide Leja chamber (37°C) and covered with a coverslip prior to analysis. The evaluated motility parameters comprised total motility (%), progressive motility (PM, %), curvilinear velocity (VCL, µm/sec), average path velocity (VAP, µm/sec), straight-line velocity (VSL, µm/sec), beat-cross frequency (BCF, Hz), and amplitude of lateral head displacement (ALH, µm). At least ten microscopic fields per sample were analyzed, and approximately 1,000 spermatozoa were tracked to calculate mean values for each parameter.

Sperm viability was assessed through the eosin–nigrosin staining method, whereby only non-viable sperm cells will have eosin–nigrosin dye uptake, while viable cells will not have the dye. Abnormalities were then classified into various types, such as head abnormalities, midpiece abnormalities, proximal droplets, detached heads, and tail abnormalities. A minimum of 200 sperm cells was assessed from 10 random microscopic fields to ascertain the percentage of viable cells.

The integrity of the plasma membrane was assessed by the hypo-osmotic swelling test (HOS) [14]. The hypo-osmotic solution was made by dissolving 0.735 gm sodium citrate (2H_2_O) and 1.351 gm fructose in 100 ml of distilled water. The test was conducted by adding 30 μl of the semen sample to 300 μl of the hypo-osmotic solution, then incubating it at 37°C for 30 min. After the incubation period, a 5 μl sample was placed on a glass slide, then observed under a light microscope at a magnification of 400x. Spermatozoa exhibiting tails curling were classified as having intact plasma membranes, whereas those with straight tails were considered to have compromised, damaged membranes. A minimum of 200 spermatozoa were assessed per sample.

**Figure 1.**
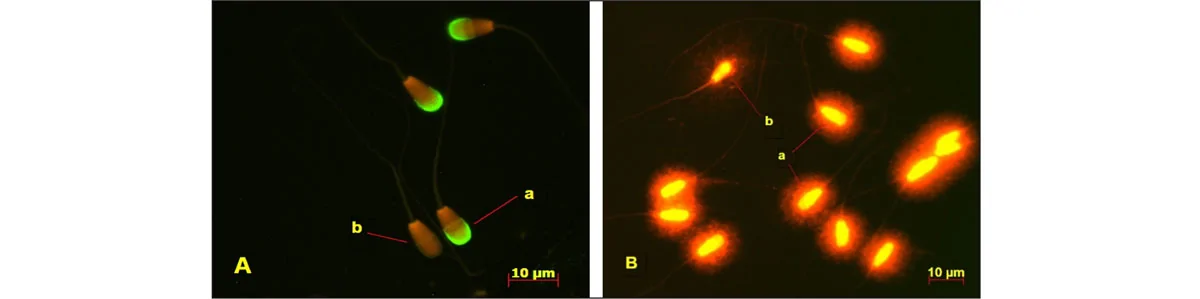
Magnification 400x, microscope fluorescence, Imager Z2, Carl Zeiss, Germany. A. Acrosome integrity. (a) Intact acrosome (green fluorescence in the acrosome), (b) Non-intact acrosome (red fluorescence). B. DNA integrity. (a) DNA intact (small or null halo), (b) DNA fragmented (large halo).

Acrosome integrity was evaluated using fluorescein isothiocyanate–peanut agglutinin (FITC-PNA) staining following the method described by Rajabi-Toustani et al. [15]. Samples were examined under a fluorescence microscope (excitation 380–420 nm), and a minimum of 200 spermatozoa were analyzed per sample. Spermatozoa cells exhibiting green fluorescence were identified as having intact acrosomes, whereas those with red fluorescence were identified as acrosome-damaged. All staining procedures were conducted in the dark to avoid light interference. Furthermore, DNA fragmentation was assessed using a modified sperm chromatin dispersion (SCD) test assay with the sperm-bos-halomax kit (Halotech DNA, SL, Spain), following the protocols of Said et al. [16] and García-Macías et al. [17]. For each slide, 200 spermatozoa were evaluated. Cells displaying a distinct halo around the sperm head were categorized to have fragmented DNA, while those lacking a halo were categorized as non-fragmented (Figure 1).

### 2.6. Data analysis

Statistical analyses of semen quality parameters were performed using independent *t*-tests and Pearson’s correlation in SPSS version 25.0 (IBM Corp., Chicago, IL, USA). Multivariate analyses, including principal component analysis (PCA) and partial least squares discriminant analysis (PLS-DA), were conducted using SIMCA version 17.0 (Sartorius Umetrics, Sweden). Prior to analysis, the data were mean-centered and scaled to unit variance. PCA was applied to characterize overall variation patterns, whereas PLS-DA was used to identify discriminant parameters between extender groups.

## 3. Results

### 3.1. Fresh semen quality

The characteristics of the fresh Bali bull semen used in this study are presented in Table 1. Semen quality from nine Bali bulls was assessed through both macroscopic and microscopic examinations. The macroscopic evaluation indicated that semen volume varied from 5 to 9 ml, with a medium to thick consistency, a milky-white color, pH values ranging from 6.1 to 6.8, and sperm concentration between 1.048 and 1.977 × 10^6^ per ml. The microscopic analysis revealed that the overall mass activity was rated as +++, total motility ranged from 82.8% to 93.84%, progressive motility from 78% to 93.32%, viability from 83.00% to 89.50%, abnormalities from 7.70% to 12.00%, and membrane integrity from 86.50% to 91.18%.

**Table 1.** The characteristics of fresh Bali bull semen.

Parameter	Bali Bulls									Mean ± SD
	Bull 1	Bull 2	Bull 3	Bull 4	Bull 5	Bull 6	Bull 7	Bull 8	Bull 9	
Volume (ml)	7.2	6	8.6	7.8	7.4	6	9	8	5	7.22 ± 1.32
Moderate	thick	thick	medium	medium	thick	medium	thick	medium	medium	medium-thick
Color	cream	cream	cream	cream	cream	cream	cream	cream	cream	cream
pH	6.2	6.2	6.5	6.5	6.1	6.2	6.2	6.8	6.4	6.34 ± 0.22
Concentration (10^9^)	1.977	1.377	1.048	1.081	1.061	1.283	1.549	1.142	1.489	1.334 ± 0.31
Mass Movement	+++	+++	+++	+++	+++	+++	+++	+++	+++	+++
Total Motility (%)	93.84	93.72	86.71	85.13	88.81	93.19	91.4	88.3	82.8	89.32 ± 3.99
Progressive mot (%)	93.32	93.08	84.48	83.59	86.74	91.99	84.8	80.9	78	86.32 ± 5.46
Viability (%)	84.00	87.00	83.00	85.50	89.50	83.00	86.09	88.46	89.58	86.24 ± 2.59
Abnormality (%)	12.00	11.50	11.00	10.78	11.47	7.70	8.00	9.80	9.60	10.21 ± 1.55
Plasma Membrane Integrity (%)	88.05	91.18	88.32	86.50	89.58	87.50	90.32	89.83	90.18	89.05 ± 1.53

Description: Bali bulls numbers 1–5 from RAIC Riau, numbers 6–9 from AIC Singosari.

### 3.2. The post-thaw semen quality

The results of post-thaw semen quality are presented in Table 2. Semen frozen with the TNL extender exhibited a significantly lower (*p* < 0.05) progressive slow motility parameter (26.25 ± 4.09%) compared to that diluted with TEY (36.78 ± 4.69%). In contrast, the progressive circular motility was notably lower (*p* < 0.05) in semen diluted with TEY (0.92 ± 0.57%) than in that with TNL (2.60 ± 1.12%). No significant differences (*p* > 0.05) were found between the two extenders, TNL and TEY, in terms of total motility, progressive motility, progressive fast motility, local motility, immotile sperm percentage, viability, abnormality, membrane integrity, acrosome integrity, or DNA fragmentation parameters. Furthermore, no significant differences (*p* > 0.05) were observed between the two extenders for other post-thaw parameters, including total motility, progressive motility, viability, abnormality rate, plasma membrane integrity, acrosome integrity, and DNA fragmentation.

**Table 2.** Post-thaw quality parameters of bull semen cryopreserved with tris–egg yolk (TEY) and tris nanoliposome (TNL) extenders.

Parameter	TEY	TNL
Total motility (%)	57.63 ± 5.56	54.54 ± 4.82
Progressive motility (%)	47.82 ± 5.51	48.40 ± 4.78
Progressive fast motility (%)	39.59 ± 7.94	46.10 ± 7.11
Progressive slow motility (%)	36.78 ± 4.69^b^	26.25 ± 4.09^a^
Progressive circular motility (%)	0.92 ± 0.57^a^	2.60 ± 1.12^b^
Local circular motility (%)	0.00 ± 0.00	0.00 ± 0.00
Local motility (%)	4.07 ± 1.58	3.11 ± 1.49
Immotile (%)	18.63 ± 5.80	21.95 ± 8.20
Viability (%)	45.45 ± 1.61	44.62 ± 1.66
Abnormality (%)	11.93 ± 1.51	10.61 ± 1.32
Plasma Membrane Integrity (%)	43.15 ± 1.67	43.59 ± 1.78
Acrosome Integrity (%)	95.40 ± 1.04	95.46 ± 0.93
DNA Fragmentation (%)	2.07 ± 0.56	2.08 ± 0.55

Description: TEY, tris egg yolk 20%; TNL, tris nano liposome (10 mg/ml). Values within the same row with different letters are significantly different (*p* < 0.05).

### 3.3. The kinematics of frozen semen

The kinematic parameters of frozen–thawed semen are presented in Table 3. No significant differences were observed between TNL and TEY diluents for all motion variables analyzed, which included velocity curvilinear (VCL), velocity straight line (VSL), velocity average path (VAP), distance curved line (DCL), distance straight line (DSL), distance average path (DAP), average lateral head displacement (ALH), beat cross frequency (BCF), wobble (WOB), linearity (LIN), and straightness (STR). The post-thaw sperm diluted with both TNL and TEY had relatively high values for VCL, VSL, VAP, ALH, BCF, WOB, LIN, and STR, which is a good indication of a good motility pattern to facilitate easy passage through the female reproductive tract, penetrate the zona pellucida, and achieve successful fertilization.

**Table 3.** The kinematics of frozen semen.

Parameters	TEY	TNL
VCL (µm/sec)	106.93 ± 7.01	112.32 ± 5.14
VSL (µm/sec)	53.04 ± 2.73	59.74 ± 3.39
VAP (µm/sec)	72.33 ± 3.56	77.20 ± 3.15
DCL (µm)	46.21 ± 3.47	49.18 ± 2.76
DSL (µm)	22.97 ± 1.23	26.25 ± 1.69
DAP (µm)	31.33 ± 1.58	33.91 ± 1.53
ALH (µm)	5.01 ± 0.73	5.92 ± 3.51
BCF (Hz)	26.71 ± 1.36	29.16 ± 1.11
WOB (%)	0.68 ± 0.02	0.68 ± 0.02
LIN (%)	0.50 ± 0.03	0.52 ± 0.02
STR (%)	0.73 ± 0.03	0.76 ± 0.03

Description: TEY, tris egg yolk 20%; TNL, tris nano liposome (10 mg/ml); VCL, velocity curved line; VAP, velocity average path; VSL, velocity straight line; DCL, distance curve linear; DSL, distance straight line; DAP, distance average path; ALH, amplitude of lateral head displacement; BCF, beat cross frequency; WOB, wobble; LIN, linearity; STR, straightness. Values within the same row with different letters are significantly different (*p* < 0.05).

### 3.4. The correlation analysis of semen quality

**Figure 2.**
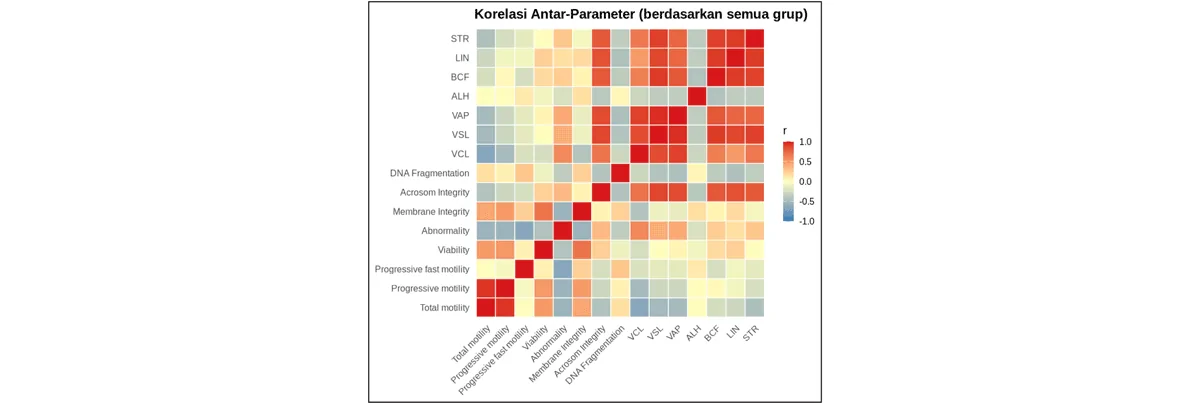
Heatmap visualization of Pearson’s correlation of sperm quality.

Correlation analysis among semen quality parameters revealed strong associations between various functional and structural attributes of spermatozoa (Figure 2). The heatmap color gradient delineates clusters of parameters revealing both positive and negative correlations, indicating close interrelationships among motility, kinematics, viability, membrane integrity, acrosome integrity, abnormality, and DNA fragmentation. A significant positive correlation (*p* < 0.05) was observed between total motility and progressive motility (*r* = 1.0), progressive motility and viability (*r* = 0.0 – 0.5), progressive motility and membrane integrity (*r* = 0.0 – 0.5), and progressive motility and acrosome integrity (*r* = 0.0 – 0.5). A significant negative correlation (*p* < 0.05) was observed between abnormality and DNA fragmentation (*r* = -0.5 to -1.0) in sperm quality after thawing. The results revealed positive correlations among kinematic parameters (*r* = 0.5 – 1.0), including VCL, VSL, VAP, BCF, and LIN, indicating that sperm exhibiting higher velocities also tended to display more linear and coordinated movement patterns. The kinematic parameters presented in Table 3 showed no statistically significant differences between semen diluted with TNL and TEY extenders, indicating comparable performance between the two extenders.

### 3.5. Multivariate analysis of sperm quality profiles

**Figure 3.**
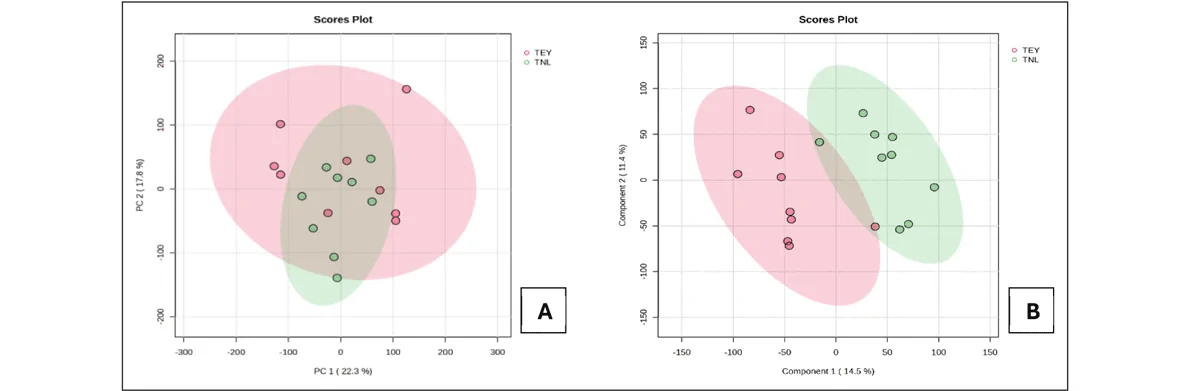
Principal component analysis and discriminant analysis of TEY and TNL extender.

Multivariate analysis was performed to assess patterns of variation and discrimination in semen quality when diluted with two types of extenders (TEY and TNL). The PCA revealed that the first two principal components (PC1 and PC2) accounted for 40.1% of the total data variance (PC1 = 22.3%; PC2 = 17.8%) (Figure 3a). The score plot indicated that TEY samples exhibited a broader distribution compared to TNL, suggesting a higher degree of variability within TEY. Conversely, TNL samples were more tightly clustered around the plot center, indicating a more uniform and stable response to dilution. Although some overlap was observed, the shift in centroid positions between the two groups highlighted distinct patterns of sperm characteristics between TEY and TNL.

The result of PLS-DA analysis indicated that there is a better separation between TEY and TNL groups. The first two components of the PLS-DA model explained 25.9% of the variance, with Component 1 contributing 14.5% and Component 2 contributing 11.4%. There is a clear boundary between TEY and TNL groups along the first component. The TEY group (in red color) is mostly concentrated on the left side of the plot, while the TNL group (in green color) is mostly concentrated on the right side of the plot (Figure 3b). This is a clear indication that there is a significant difference in sperm quality profile between these two groups. The overlapping region is very narrow, which indicates that these samples can be correctly classified.

## 4. Discussion

### 4.1. Fresh semen quality

The fresh Bali bull semen used in this study exhibited acceptable quality and complied with the standards established by the Indonesian National Standard for frozen bull semen (SNI 4869.1-2021), which requires a minimum progressive sperm motility of 70% and a maximum sperm abnormality rate of 20% [18]. The results of the macroscopic examination indicated that the volume, consistency, color, odor, and pH of the semen were within the normal physiological limits, consistent with the characteristics of high-quality bovine semen, according to Arifiantini [19]. The results of the microscopic examination further confirmed the high quality of the samples, showing that the viability of the sperm was more than 80%, meeting the requirements [20].

The proportion of spermatozoa exhibiting intact plasma membranes exceeded 60%, a level considered optimal for maintaining sperm fertilizing capacity [21]. In addition, the percentage of abnormal spermatozoa remained below 20%, thereby fulfilling the threshold established by the national standard [18]. These findings indicate that the fresh semen possessed strong physiological integrity and was suitable for subsequent cryopreservation procedures. Furthermore, high levels of motility, viability, and membrane integrity are recognized as key indicators of semen quality following freezing.

### 4.2. The post-thaw semen quality

The findings on post-thaw semen quality using two different extenders (TEY and TNL) revealed that sperm in the TNL extender exhibited fewer slow progressive movements and a slightly higher proportion of circular progressive motion compared to sperm in TEY. This can be attributed to the lower viscosity of the TNL extender, which offers a clearer medium and facilitates more precise motility analysis using CASA systems. Similar results with previous studies by Hameed et al. [22] and Ahmadi et al. [23], where variations in extender viscosity and osmolarity significantly affected sperm motility patterns during CASA evaluation. This suggests that both extenders can maintain the structural and functional integrity of spermatozoa during cryopreservation and thawing.

### 4.3. The kinematics of frozen semen

VCL values below 150 µm/sec indicated that sperm hyperactivation had not occurred, which was further supported by the high level of acrosome integrity, suggesting the absence of premature capacitation. The comparable quality outcomes observed between treatments indicate that the TNL extender was effective in maintaining the post-thaw semen quality of Bali bulls. In particular, the progressive motility rate exceeded 40%, meeting the minimum quality requirement for frozen semen established by the Indonesian National Standard (SNI) [18]. The ability of the TNL extender to preserve sperm motility and membrane stability may be attributed to its optimized composition of cryoprotectants, osmolytes, and buffering agents, which help mitigate cold shock and oxidative stress during the freezing process [24, 25]. Previous studies have highlighted the importance of extender formulation, particularly the balanced inclusion of lipids and sugars, in enhancing sperm resilience during cryopreservation by maintaining mitochondrial function and plasma membrane integrity [26]. The findings of the present study, therefore, emphasize the critical role of extender composition in preserving sperm functional integrity following the thawing process.

This study also showed that TEY extenders can be effectively replaced by TNL formulations for bovine semen cryopreservation. Egg yolk has long been recognized as a common cryoprotectant used during the freezing and thawing of spermatozoa in most mammalian species [27]. The benefits of using egg yolk in semen cryopreservation are mainly due to its protective factors, which shield spermatozoa from cold shock damage, and its storage-related components, which help maintain sperm motility, viability, and fertilizing capacity [28]. However, egg yolk has several drawbacks, including compositional inconsistencies, interference of yolk granules with sperm motility, and potential microbial contamination. It has been reported that egg yolk particles in extenders may be mistakenly counted as sperm heads when analyzed using CASA, leading to overestimation of sperm concentration and inaccurate motility assessment [29]. In this study, sperm diluted in the TNL extender appeared optically clearer than those diluted in the traditional TEY extender, facilitating more accurate sperm evaluation.

### 4.4. The correlation analysis of semen quality

Sperm motility is a crucial factor in fertility, as it facilitates the movement of spermatozoa through the female reproductive tract and ensures successful fertilization [30]. Saadeldin et al. [31] reported that exogenous phospholipids in plant-based and liposome-based extenders, which consist of various lipid species, can protect spermatozoa by reversibly binding to sperm lipids and phospholipids and integrating with the sperm plasma membrane. This interaction stabilizes the membrane structure during the freeze–thaw process, thereby minimizing cryo-induced damage. Additionally, lecithin, a vital component of nanoliposome extenders, shows greater tolerance to osmotic stress during cryopreservation, aiding in the preservation of the sperm plasma membrane’s integrity and functionality [32]. As a result, the post-thaw semen quality parameters in the TNL extender were not significantly different from those in the TEY extender. Luna-Orozco et al. [33] reported that sperm diluted with plant-derived liposomal extenders exhibited improved kinematic characteristics compared with those diluted in egg yolk–based extenders. In contrast, Duracka et al. [34] found that TEY-based extenders resulted in higher total and progressive motility than lecithin-based extenders. These discrepancies may be associated with species-specific differences in sperm membrane composition and their interactions with extender components. Nevertheless, the findings of the present study suggest that TNL-based extenders can serve as an efficient, cleaner, and biosecure alternative to conventional TEY-based extenders without compromising the quality of frozen–thawed sperm.

These bioactive molecules have been widely utilized as alternative cryoprotective components to TEY-based extenders in commercial applications and have demonstrated comparable *in vitro* outcomes across several species, including cattle [35], horses [36], and buffaloes [37]. In the present study, the absence of significant differences in sperm quality parameters between TEY and TNL extenders indicates that both formulations provide effective protection against cryoinjury and are capable of preserving sperm functional integrity during the freeze–thaw process. The comparable performance of TNL and TEY extenders in maintaining sperm kinematic parameters is of both biological and practical significance. Biologically, the preservation of motility parameters such as VCL, VSL, and BCF at levels comparable to those observed with TEY indicates that the plant-derived TNL extender effectively supports mitochondrial activity and flagellar function during cryopreservation. These findings support the concept that efficient energy metabolism and the maintenance of membrane structural integrity are critical determinants of post-thaw sperm functionality and fertilization potential [38].

The structural stability of plant-derived phospholipids and fatty acids may protect spermatozoa from oxidative damage and lipid peroxidation during the freezing process, thereby enhancing sperm viability [39]. The application of TNL extenders also offers several advantages in reproductive biotechnology, particularly in semen cryopreservation. Unlike TEY-based extenders, plant-derived formulations minimize the risk of microbial contamination associated with animal-derived components, thereby improving biosecurity [40]. In addition, the more consistent composition of plant-based extenders facilitates better standardization, especially when automated semen processing systems are employed. Collectively, these findings highlight the potential of TNL as an economically feasible and biosecure alternative extender capable of maintaining sperm viability during cryopreservation, comparable to conventional extenders used in artificial insemination and genetic conservation programs.

The higher BCF values observed in TNL-treated samples suggest that the TNL extender may be more effective in preserving flagellar structural integrity and supporting energy metabolism through enhanced ATP production, thereby maintaining a stable frequency of sperm tail beating. This observation is consistent with the findings of Murphy et al. [41], who reported improved sperm motility when plant-based extenders were used compared with conventional egg yolk–based diluents. Plant-derived extenders typically contain natural phosphatidylcholine and essential fatty acids, such as stearic, oleic, and palmitic acids, which have been shown to contribute to the maintenance of sperm membrane structural integrity.

### 4.5. Multivariate analysis of sperm quality profiles

Correlation analysis of semen quality parameters revealed strong associations among several functional and structural attributes of spermatozoa, reflecting the integrative nature of sperm cell physiology. The heatmap clustering pattern further demonstrated both positive and negative correlations among motility, kinematic parameters, viability, membrane integrity, acrosome integrity, abnormality, and DNA fragmentation, highlighting the interdependent relationships among key indicators of semen quality. A strong positive correlation was observed between total motility and progressive motility (*r* = 1.0), indicating that samples with higher total motility also exhibited greater progressive motility. This relationship reflects coordinated flagellar activity and efficient energy metabolism, both of which are essential for optimal sperm function and fertilization potential [42]. Additionally, significant positive correlations among kinematic parameters, including VCL, VSL, VAP, BCF, and LIN, suggest that spermatozoa with higher velocities also tend to exhibit more linear and efficient movement patterns. This pattern supports previous findings that sperm velocity and linearity are key determinants of fertilization potential [43].

The positive correlations among membrane integrity, acrosome integrity, motility, and viability highlight the critical role of structural integrity in determining the functional competence of spermatozoa. Sperm cells with intact plasma membranes and acrosomes are more capable of maintaining motility and resisting osmotic and oxidative stress during processing and cryopreservation [44]. These findings further support the concept that membrane and acrosomal integrity represent not only morphological characteristics but also key determinants of the metabolic activity and mechanical functionality of sperm cells. Conversely, sperm abnormality and DNA fragmentation exhibited strong negative correlations with motility, viability, and membrane integrity. Increased levels of morphological abnormalities and DNA damage were associated with reduced sperm quality. These findings are consistent with the concept that both structural and genomic integrity are essential determinants of sperm functionality [45].

These findings highlight the importance of multivariate correlation analysis as a robust approach for comprehensively evaluating sperm performance under different extender treatments. The PCA results indicated that the first principal component was primarily driven by sperm motility and velocity parameters. In contrast, the second principal component was mainly associated with structural attributes, including membrane integrity and sperm abnormalities. These observations are consistent with the correlation analysis, which demonstrated positive associations between motility parameters and structural integrity, as well as negative relationships between sperm abnormalities and functional performance. Similar findings have been reported in previous studies, indicating that sperm motility serves as a reliable indicator of cellular health and functional status [46]. The integrity of the plasma membrane plays a crucial role in maintaining ionic balance and enzymatic activity, both of which are essential for supporting flagellar motility [47]. Furthermore, morphological abnormalities have been associated with defects in mitochondrial structure and the axoneme, which can impair sperm motility and ultimately reduce fertilization potential [48].

The distinct distribution pattern observed in the PLS-DA plot further indicates the biological influence of extender composition on sperm functional characteristics. Semen processed with the TNL extender exhibited greater consistency in quality, particularly in terms of membrane integrity and motility, whereas samples extended with TEY displayed a broader range of variability. These differences may be attributed to variations in osmotic balance, cryoprotective components, and buffering capacity between the two extenders, which can directly affect sperm metabolism and structural stability during cryopreservation [49]. The combined interpretation of the PCA and PLS-DA results further supports the superior protective capacity of the TNL extender in preserving sperm quality, contributing to greater stability and uniformity in spermatozoa. These findings underscore the critical role of extender formulation in enhancing sperm resilience during dilution and cryopreservation, with TNL demonstrating greater efficiency in maintaining post-thaw semen viability [12, 50].

Although the present study produced promising findings, it is important to acknowledge that the evaluation was limited to immediate post-thaw *in vitro* assessments and did not include analyses of sperm longevity during extended incubation or reproductive performance *in vivo*. Consequently, potential differences in the effects of the extenders on post-thaw cellular persistence and stability over prolonged incubation periods were not fully investigated. Therefore, further studies incorporating long-term *in vitro* functional assessments and fertility trials are recommended to comprehensively validate the biological efficacy of the TNL extender.

## 5. Conclusions

Both TEY and TNL extenders preserved post-thaw semen quality and sperm kinematics characteristics in Bali bulls. Multivariate analyses (PCA and PLS-DA) identified sperm motility and membrane integrity as key determinants of overall semen quality, with TNL demonstrating more consistent and stable responses. These findings indicate that TNL provides cryoprotective performance comparable to that of the conventional TEY extender while offering additional advantages in terms of compositional standardization and improved biosecurity. Therefore, TNL represents a promising and sustainable alternative extender for bovine semen cryopreservation and may support its broader application in cattle breeding programs.

## Data Availability

The data presented in this study are available from the corresponding author upon reasonable request.

## List of abbreviations

AI, artificial insemination; ALH, average lateral head displacement; BCF, beat cross frequency; CASA, computer-assisted sperm analysis; DMSO, dimethyl sulfoxide; DMF, dimethylformamide; DAP, distance average path; DCL, distance curved line; DSL, distance straight line; LIN, linearity; PLS-DA, partial least squares–discriminant analysis; PCA, principal component analysis; SOP, standard operating procedures; STR, straightness; TEY, Tris egg yolk-based extenders; TNL, Tris-nano-liposome-based extenders; VAP, velocity average path; VCL, velocity curvilinear; VSL, velocity straight line; WOB, wobble.

## References

[1] Iskandar H, Andersson G, Sonjaya H, Arifiantini RI, Said S, Hasbi H (2023). Protein identification of seminal plasma in Bali bull (*Bos javanicus*). Animals.

[2] Ministry of Agriculture (2010).

[3] Kapa M, Henuk YL, Hasnudi, Suyadi S (2018). Contribution of local beef cattle production on farmer’s income in the dryland farming of Kupang Regency, Indonesia. IOP Conf Ser Earth Env Sci.

[4] Zuidema D, Kerns K, Sutovsky P (2021). An exploration of current and perspective semen analysis and sperm selection for livestock artificial insemination. Animals.

[5] Permana RGS, Puspitadesy SA, Nareswari A, Ediyono S (2024). Influence of method thawing to success artificial insemination of cows. Int J Life Sci Agric Res.

[6] Suarez AU, Vallejo V, Restrepo G (2025). Establishing a sperm quality index for frozen–thawed bovine semen based on sperm motility, viability and capacitation traits. Reprod Domest Anim.

[7] Fitriana SB, Maghfiroh NA, Baity AN, Diatmono DFF, Prihantoko KD, Bintara S (2025). Effect of different thawing methods on frozen semen characteristics and DNA damage of Indonesian Simmental bull. Pak J Agric Res.

[8] Bustani GS, Baiee FH (2021). Semen extenders: An evaluative overview of preservative mechanisms of semen and semen extenders. Vet World.

[9] Silva ECB, Cajueiro JFP, Silva SV, Vidal AH, Soares PC, Guerra MMP (2012). *In vitro* evaluation of ram sperm frozen with glycerol, ethylene glycol or acetamide. Anim Reprod Sci.

[10] Jalal MP, Mehdipour M, Sharifi SD, Farhadi R (2025). *Thymus vulgaris* extract as a potent cryoprotectant enhancing antioxidant defense and sperm quality in roosters. Poult Sci.

[11] Yildiz C, Ottaviani P, Law N, Ayearst R, Liu L, McKerlie C (2013). Effects of cryopreservation on sperm quality and fertility in mammals. Reprod Fertil Dev.

[12] Gunawan M, Karja NWK, Setiadi MA, Kaiin EM, Said S, Iskandar H (2025). Development and evaluation of soy lecithin-derived nanoliposomes as a plant-based alternative to egg-yolk extender for Ongole-grade bull semen cryopreservation. Vet World.

[13] Khalili WA, El-Rais MS, Hegazy MM, Hassan MAE, El-Raghi AA, El-Moghazy MM (2025). The effect of metallic nanoparticles supplementation in semen extender on post-thaw quality and fertilizing ability of Egyptian buffalo (*Bubalus bubalis*) spermatozoa. Biol Trace Elem Res.

[14] Maulana T, Said S, Arifiantini RI, Jakaria J, Gunawan A (2024). The frozen-thawed sperm protein of Indonesian Toraya buffaloes is significantly associated with sperm kinematics, acrosome integrity, and mitochondrial membrane potential. J Adv Vet Anim Res.

[15] Rajabi-Toustani R, Akter QS, Almadaly EA, Hoshino Y, Adachi H, Mukoujima K (2019). Methodological improvement of fluorescein isothiocyanate peanut agglutinin (FITC-PNA) acrosomal integrity staining for frozen-thawed Japanese Black bull spermatozoa. J Vet Med Sci.

[16] Said S, Diansyah AM, Rahayu JD, Maulana T, Gunawan M, Kaiin EM (2025). A comparative analysis of semen quality traits and sperm kinematic parameters in relation to fertility prediction in Murrah buffaloes. J Adv Vet Anim Res.

[17] Garcia-Macias V, de Paz P, Martinez-Pastor F, Alvarez M, Gomes-Alves S, Bernardo J (2007). DNA fragmentation assessment by flow cytometry and sperm-Bos-halomax (bright-field microscopy and fluorescence microscopy) in bull sperm. Int J Androl.

[18] BSN (Badan Standardisasi Nasional) (2021).

[19] Arifiantini RI

[20] Anwar K, Thaller G, Saeed-Zidane M (2024). Sperm-borne mitochondrial activity influenced by season and age of Holstein bulls. Int J Mol Sci.

[21] Rizal M, Syarifuddin NA, Sumantri I, Wahdi A, Riyadhi M, Rizqiana S (2025). Cryopreservation of Boer crossbreed goat semen with Andromed extender and *Moringa* leaf extract. Acta Vet Indones.

[22] Hameed N, Zubair M, Ahmad N, Durrani AZ, Khan MIUR (2023). Effect extender type and storage time on sperm quality parameters of Kail ram semen stored at 5°C. Trop Anim Health Prod.

[23] Ahmadi MHN, Mirzaei A, Divar MR, Derafshani F (2025). Optimising filtered egg yolk extenders for long-term cold preservation of ram sperm: effects of osmolarity and egg yolk concentration. Reprod Domest Anim.

[24] Akhter S, Ansari MS, Andrabi SMH, Ullah N, Qayyum M (2012). Effect of soybean-based extenders on sperm parameters of Nili-Ravi buffalo (*Bubalus bubalis*) bull semen during liquid storage. Pak J Zool.

[25] Hungerford A, Bakos HW, Aitken RJ (2022). Sperm cryopreservation: Current status and future developments. Reprod Fertil Dev.

[26] Ruiz ED, Bermejo JVD, Jurado JML, Gonzales FJN, Arbulu AA, Guzman JFB (2024). Effect of supplementation of a cryopreservation extender with Pectoliv30 on post-thawing semen quality parameters in rooster species. Antioxidants.

[27] Larasati MD, Lestari SW, Pangestu M, Hestiantoro A, Wasian G (2024). Modification of cryoprotectants on sperm cryopreservation: A study of embryo development. J Adv Vet Anim Res.

[28] Aboagla E (2004). Effects of egg yolk during the freezing step of cryopreservation on viability of goat spermatozoa. Theriogenology.

[29] Layek SS, Mohanty TK, Kumaresan A, Parks JE (2016). Cryopreservation of bull semen: Evolution from egg yolk-based to soybean lecithin-based extenders. Anim Reprod Sci.

[30] Chakraborty S, Saha S (2022). Understanding sperm motility mechanisms and the implication of sperm surface molecules in promoting motility. Middle East Fertil Soc J.

[31] Saadeldin IM, Khalil WA, Alharbi MG, Lee SH (2020). The current trends in using nanoparticles, liposomes, and exosomes for semen cryopreservation. Animals.

[32] Bodu M, Hitit M, Greenwood OC, Murray RD, Memili E (2025). Extender development for optimal cryopreservation of buck sperm to increase reproductive efficiency of goats. Front Vet Sci.

[33] Luna-Orozco JR, Gonzalez-Ramos MA, Calderon-Leyva G, Gaytan-Aleman LR, Arellano-Rodriguez F, Angel-Garcia O (2019). Comparison of different diluents based on liposomes and egg yolk for ram semen cooling and cryopreservation. Iran J Vet Res.

[34] Duracka M, Benko F, Kacaniova M, Tvrda E (2024). Comparative study: Efficacy of egg-yolk soy lecithin-based diluent in preservation of chilled bovine semen-Bacteriology and sperm quality. Czech J Anim Sci.

[35] Aires VA, Hinsch KD, Mueller-Schoesser F, Bogner K, Mueller-Scloesser S, Hinsch E (2003). *In vitro* and *in vivo* comparison of egg yolk-based and soybean lecithin-based extenders for cryopreservation of bovine semen. Theriogenology.

[36] Pillet E, Labbe C, Batellier F, Duchmamp G, Beaumal V, Anton M (2012). Liposomes as an alternative to egg yolk in stallion freezing extender. Theriogenology.

[37] Kumar R, Atreja SK (2012). Effect of incorporation of additives in tris-based egg yolk extender on buffalo (*Bubalus bubalis*) sperm tyrosine phosphorylation during cryopreservation. Reprod Domest Anim.

[38] Xue SH, Xu BB, Yan CC, Zhang JX, Su R (2025). Sperm membrane stability: In-depth analysis from structural basis to functional regulation. Vet Sci.

[39] Liu X, Zeng T, Zhang E, Bin C, Liu Q, Wu K (2025). Plant-based bioactives and oxidative stress in reproduction: anti-inflammatory and metabolic protection mechanisms. Front Nutr.

[40] Sharaf AE, El-Harairy MA, Khalifa EI, Abdelnour SA, Khalil WA (2026). The shift toward antibiotic-free semen extenders: Innovative uses of natural substances in ruminant sperm cryopreservation. Reprod Domest Anim.

[41] Murphy EM, O’Meara C, Eivers B, Lonergan P, Fair S (2018). Comparison of plant- and egg yolk-based semen diluents on *in vitro* sperm kinematics and *in vivo* fertility of frozen-thawed bull semen. Anim Reprod Sci.

[42] Sharma A, Anand M, Pareek S, Yadav S, Yadav B, Dhariya R (2025). Semen extenders in caprine reproduction: Composition, application and future prospects. Int J Vet Sci Anim Health.

[43] Hadlow JH, Lymbery RA, Evans JP (2022). Density-dependent patterns of multivariate selection on sperm motility and morphology in a broadcast spawning mussel. Ecol Evol.

[44] Ozimic S, Ban-Frangez H, Stimpfel M (2023). Sperm cryopreservation today: Approaches, efficiency, and pitfalls. Curr Issues Mol Biol.

[45] Tesarik J, Tesarik RM (2025). Sperm-derived dysfunction of human embryos: Molecular mechanisms and clinical resolution. Int J Mol Sci.

[46] Barbagallo F, Vignera SL, Cannarella R, Aversa A, Calogero AE, Condorelli RA (2020). Evaluation of sperm mitochondrial function: A key organelle for sperm motility. J Clin Med.

[47] Wang Y, Fu X, Li H (2025). Mechanisms of oxidative stress-induced sperm dysfunction. Front Endocrinol.

[48] Raad MV, Firouzabadi AM, Niaki MT, Henkel R, Fesahat F (2024). The impact of mitochondrial impairments on sperm function and male fertility: A systematic review. Reprod Biol Endocrinol.

[49] Saratsi A, Samartzi F, Tsiokos D, Theodosiadou EK, Panagiotidis I, Ligda C (2024). Effect of three commercially available extenders containing phospholipids of different sources on Skopelos buck liquid-stored sperm quality. Vet Sci.

[50] Buyukleblebici S, Tuncer PB, Bucak MN, Eken A, Sariozkan S, Tasdemir U (2014). Cryopreservation of bull sperm: Effects of extender supplemented with different cryoprotectants and antioxidants on sperm motility, antioxidant capacity and fertility results. Anim Reprod Sci.

